# Environmental and health risk assessments of genetically modified eucalypts in Brazil

**DOI:** 10.1186/1753-6561-5-S7-O57

**Published:** 2011-09-13

**Authors:** Giancarlo Pasquali, Leandro V Astarita, Eliane R Santarém, Rogério Margis, Rafael Roesler, Eduardo Cassel, Aline M Lucas, Juliana Gerhardt, Maria N M Lima, Joseane B Carvalho, Karen A Freitas, Rochele P Kirch

**Affiliations:** 1 UFRGS, Porto Alegre, RS, Brazil; 2 PUCRS, Porto Alegre, RS, Brazil; 3 PUCRS, Brazil; 4 UFRGS & Vitatec, Porto Alegre, RS, Brazil; 5 UFRGS & NeuroAssay, Porto Alegre, RS, Brazil; 6 NeuroAssay, Brazil; 7 Vitatec, Brazil; 8 UFRGS, Brazil

## 

Motivated by the Brazilian Ministry of Agriculture, Farming and Supply (MAPA), we created in 2009 the “Collaborating Center in Agriculture Defense Relative to the Biosafety of Genetically Modified Eucalypts” (Project “CDA *Eucalyptus*”) in order to collect information and conduct research to assess the biosafety of GM eucalypts in the Brazilian context. The Normative Resolution Nr. 5 of the National Biosafety Technical Commission (CTNBio) is the official document presenting all information needed to propose the commercial release of GMOs in Brazil. Based on this document and along with the personnel of the Suzano Paper & Cellulose Co., we conducted a series of experiments with GM and non-GM eucalypts planted in a test field in the state of São Paulo to start collecting the necessary information. Two independent groups of transgenic plants, harboring two different transgene constructions along with non-GM control plants are being assayed. The genetic traits, the identity or names of the transgenes as well as the identity of each tree individual will not be revealed due to intellectual property request still pending. Each group of plants was represented by four independent events in triplicates (2 groups x 4 events x 3 clonal trees + 3 non-GM clonal trees), therefore totaling 27 individuals under analysis. Samples were identified by random numbers and all assays were conducted in a simple-blind or a double-blind fashion. Tests concluded until now included (i) the detection of transgene regulatory sequences in purified DNA samples by conventional PCR and RT-qPCR, confirming the expected sampling conducted; (ii) extraction, chemical characterization and analysis of the antifungal effects of essential (volatile) oils extracted from leaves; (iii) pollen germination *in vitro*; (iv) flower morphology; (v) seed production; (vi) initial seedling development; (vii) leaf allelopathy; (viii) measurements of total phenolic compounds in leaves and roots; and (ix) effects of leaf extracts on the viability of human colon cells. All results obtained from experiments (ii) to (ix) revealed no statistical differences between GM- and non-GM-derived samples. A second round of experiments will be conducted to confirm these results. Proteomic and transcriptomic profiling of GM and non-GM trees are under analysis, as well as a series of experiments that include the chemical, nutritional and biological analysis of honey samples derived from bee hives located in fields of GM *versus* non-GM plants; and bee (*Apis mellifera*) population dynamics.

